# The Interaction of Cue Type and Its Associated Behavioral Response Dissociates the Neural Activity between the Perirhinal and Postrhinal Cortices

**DOI:** 10.1523/ENEURO.0065-22.2022

**Published:** 2022-04-26

**Authors:** Heung-Yeol Lim, Jae-Rong Ahn, Inah Lee

**Affiliations:** 1 Department of Brain and Cognitive Sciences, Seoul National University, Seoul 08826, Korea; 2 Department of Biology, Tufts University, Medford, Massachusetts 02155

**Keywords:** entorhinal cortex, episodic memory, hippocampus, perirhinal cortex, postrhinal cortex, spatial memory

## Abstract

The perirhinal cortex (PER) and postrhinal cortex (POR) in the medial temporal lobe are commonly described as two distinct systems that process nonspatial and spatial information, respectively. Recent findings suggest that the two regions exhibit functional overlap when processing stimulus information, especially when associative responses are required in goal-directed behavior. However, we lack the neural correlates of this. In the current study, we recorded spiking activities for single units of the PER and POR as rats were required to choose a response associated with the identity of a visual object or scene stimulus. We found that similar proportions of cells fired selectively for either scene or object between the two regions. In the PER and POR, response-selective neurons showed higher contrast for different responses than stimulus-selective cells did for stimuli. More cells fired selectively for specific choice response in the POR than in the PER. The differential firing patterns of the PER and POR were best explained when the stimulus and response components were considered together: Stimulus-selective cells were modulated more by the response in the POR than in the PER, whereas response-selective cells in the PER were modulated more by object information than by scenes. Our results suggest that in a goal-directed memory task, the information processing in the PER and POR may be dynamically modulated not only by input stimulus information but also by the associated choice behavior and stimulus–response interaction.

## Significance Statement

The perirhinal (PER) and postrhinal cortex (POR) are two major gateways of information processing in the medial temporal lobe, but their exact roles are still elusive. We recorded spiking activities of single units from the PER and POR while rats performed a goal-directed task using objects and scenes as cues. We report that the two regions are hardly differentiated just based on the stimulus type, and that the neural firing patterns are best understood when considering the type of stimulus and choice response together. Our results stand in contrast to the traditional theory that has emphasized the stimulus type only to dissociate the PER and POR and illustrate how task demand dynamically influences the information processing in the parahippocampal region.

## Introduction

In the medial temporal lobe (MTL), the perirhinal cortex (PER) and postrhinal cortex (POR) can be considered gateways where sensory-perceptual information is transformed into mnemonic information before being fed to downstream regions, such as the entorhinal cortex and hippocampus (Burwell, 2000; Witter et al., 2000). Traditionally, the PER and POR have been considered to send fairly segregated projections to the lateral entorhinal cortex (LEC) and medial entorhinal cortex (MEC), respectively (Suzuki and Amaral, 1994; Naber et al., 1997; Burwell and Amaral, 1998a). In support of the anatomic literature, some significant physiological differences have been reported between the MEC and LEC (Hafting et al., 2005; Savelli et al., 2008; Solstad et al., 2008; Kropff et al., 2015). Furthermore, behavioral studies directly comparing the roles of the PER and POR in a spontaneous object exploration task showed that lesions in the PER and POR produced deficits in showing a preference for a novel object (i.e., nonspatial memory deficit) and a novel position (i.e., spatial memory deficit), respectively, adding up to the parallel functional segregations of the PER and POR functions within the MTL (Norman and Eacott, 2005).

However, recent anatomic findings have shed new light on this notion of segregated pathways of the MTL by showing that the POR in addition to the PER project heavily to the LEC (Doan et al., 2019). Furthermore, the POR and PER are interconnected reciprocally (Burwell and Amaral, 1998b; Lavenex et al., 2004; Burke et al., 2018). It is also unclear whether the previous experimental evidence supports the clear physiological differentiation between the PER and POR (Furtak et al., 2012; Bos et al., 2017). Also, the PER and POR are not dissociated very well in a visual scene memory task, compared with the LEC and MEC (Park et al., 2017). Considering these findings, the traditional view needs to be modified to guide future experimental work and reinterpret the results from prior studies that showed segregated functions of the PER and POR (Manns and Eichenbaum, 2006; Knierim et al., 2014). For this purpose, the neural correlates of the PER and POR need to be directly compared within the same animals that perform in a common task, but very few studies have done so.

In rodents, the relative lack of physiological studies on the PER and POR, compared with the LEC and MEC, limits our ability to fully understand information processing in the hippocampal memory systems. In humans, in contrast, differential neural correlates have been widely studied between the PER and the parahippocampal cortex (PHC), which is the primate homolog of the POR. According to the human experimental literature, the PER and PHC process different categories of visual stimuli when subjects judge the familiarity or category of a visual image (Robin et al., 2019). For example, human fMRI studies have consistently found that the PER is reliably activated by “objects,” which can be thought of as nonspatial information, whereas the PHC is mostly activated by “visual scenes” that provide spatial contextual information (Litman et al., 2009; Duarte et al., 2011; Berron et al., 2018). Multivariate pattern analysis of fMRI signals also revealed that the PER is better at classifying objects than scenes and vice versa for the PHC (Diana et al., 2008; Martin et al., 2013; Huffman and Stark, 2014; Kafkas et al., 2017). Single-unit neural activity recordings in human subjects have also provided evidence for the presence of scene-selective neurons in the PHC (Mormann et al., 2017).

The prior studies largely examined the functional dissociations between the PER and PHC by using the “type of stimulus content” as the criterion, without considering any associated behavior. Recent studies from our laboratory have demonstrated that, in a goal-directed memory task, the parahippocampal regions show critical information-processing differences between the stimulus type and the associated behavioral response (Park et al., 2017; Yoo and Lee, 2017; Lee et al., 2021). In one study, rats were required to make a goal-directed choice response associated with the identity of a visual scene. The following two tasks were asked of the same rats: choose the left or right arm of a T-maze (i.e., navigational response), and dig sand in a jar or push the jar (i.e., non-navigational response). When different stimulus types (scene or object) were associated with different types of responses (navigational or non-navigational choice), we observed a functional double dissociation between the MEC and LEC: inactivating the MEC produced deficits only when navigational responses were required, whereas inactivating the LEC led to deficits in the non-navigational task (Yoo and Lee, 2017). Interestingly, when the roles of the PER and POR were tested in the same task, no such interaction between the stimulus and response type was found between the two regions (Park et al., 2017). It was also demonstrated that inactivation of the PER impaired performance most severely when objects were used as cues, although the same animals also exhibited deficits when scenes were used as cues. Meanwhile, inactivation of the POR clearly impaired performance in the scene-cued task and also affected performance in the object-cued task if object cues were presented only visually. Together, these findings suggest that the PER and POR may not be clearly dissociated only based on information content (e.g., object, scene).

In the current study, we investigated further whether the PER and POR could be dissociated functionally by recording single units from them in rodents performing a visual scene and object memory (VSOM) task. The VSOM task was similar to the task used in a previous study (Park et al., 2017), with modification to accommodate the electrophysiological experimental setup used to record the spiking activities of single units while the animal experienced objects and scene stimuli interchangeably within the same recording session. We report here that similar proportions of object-selective and scene-selective neurons are found in the PER and POR, but the POR contains more neurons that exhibit selective firing for a given choice response. Importantly, the stimulus-selective neurons in the POR were more significantly modulated by the response factor than those in the PER. Furthermore, the response-selective neurons in the PER fired more specifically for object stimuli compared with those in the POR. Our findings suggest that the neural correlates of both stimulus and response should be considered together when one seeks to fully understand the neural mechanisms of goal-directed mnemonic behavior in the PER and POR.

## Materials and Methods

### Subjects

Eight male Long–Evans rats (8 weeks old) were obtained and individually housed in a temperature- and humidity-controlled animal colony. Rats were allowed free access to food pellets and water for 1 week, but during experimental sessions, food was limited to two or three pellets (6–10 g) per day to maintain them at ∼80% of their free-feeding body weight. Rats were maintained on a 12 h light/dark cycle (lights on at 8:00 A.M.), and all experiments were conducted in the light phase. All animal procedures were performed in accordance with the regulations of the International Animal Care and Use Committee of the Seoul National University.

### Behavioral apparatus

**Figure 1.**
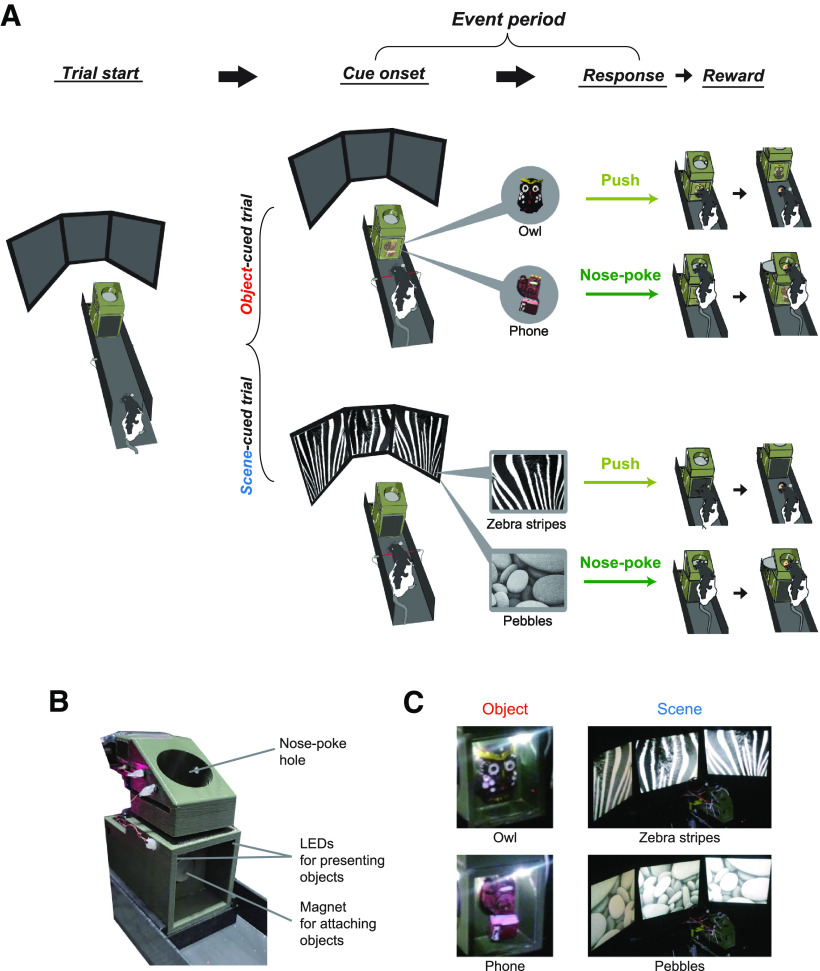
Visual scene and object memory (VSOM) task. ***A***, Illustration of the apparatus, stimuli, and task structure. A scene (zebra or pebble patterns) or object (phone or owl) was pseudorandomly presented when the rat activated the optic sensor (Cue onset). The object stimulus was attached to the front opening of the response box via a magnet and partitioned with a transparent acrylic blocker. The scene stimulus was displayed through an array of LCD monitors that surrounded the linear track. The rat was required to either push or nose-poke the response box to obtain a food reward (Response). The time from the cue onset to the choice response was defined as an event period. ***B***, A picture of the response box used for object presentation and choice response (i.e., push or nose-poke). Food reward was placed either under the response box (for push response) or in the nose-poke hole (for nose-poke response). ***C***, Stimuli used in the VSOM task. For object presentation, an owl or phone object was attached to the response box. Scenes (zebra stripes or pebbles) were presented on the surrounding three LCD monitors.

The apparatus consisted of an elevated linear track (46 × 7.5 cm; 94 cm above the floor) with a guillotine door-operated start box (22 × 16 × 31 cm) attached at the end of the track. A circular food well (diameter, 2 cm) was recessed on the floor of the track and covered with a custom-built response box (13 × 6 × 13 cm; Fig. 1*B*). A proximity sensor was built into the center of the food well to detect the displacement of the response box (i.e., for push response). A rectangular opening (6 × 5 cm) was made on the front panel of the response box so that an object stimulus could be attached with a magnet. LEDs were attached at the upper left and right corners of the front opening of the box and were turned on when the rat activated the optical sensor positioned 2 cm in front of the box. The response box was surrounded by an array of LCD monitors (17 inch) that provided visual scene images. The top of the response box was beveled (30°) and contained a recessed circular hole (diameter, 4 cm) on its surface. The upper hole was equipped with an infrared sensor that was blocked with a motorized acrylic blocker. The sensor was activated, and the hole was opened when a rat put its muzzle into the hole (i.e., for nose-poke response). Rats gained access to a food reward (a quarter piece of Froot-Loops cereal, Kellogg’s) on successful execution of a response (i.e., push or nose-poke) associated with the presented stimulus. An ATmega 128 board controlled the sensors, lights for object presentation, and motors. A custom-built MATLAB program was used to control monitors for scene presentation through communication with the ATmega 128 board. The apparatus was surrounded by circular black curtains, and all experiments were performed in the dark. A masking white noise (80 dB) was played through an in-room loudspeaker throughout all experiments.

### Visual scene and object memory task

Rats were trained in a visual recognition memory task with a pair of three-dimensional objects (refrigerator magnets; Owl, 4.2 × 3 × 2 cm; Phone, 5.5 × 3 × 1.5 cm) and visual scene images (zebra stripes and pebble pattern; Fig. 1*C*). In object trials, one object stimulus was attached to the front opening of the response box with a magnet (Fig. 1*A*,*B*). The object was then partitioned with a transparent sliding acrylic blocker to limit the sampling modality to vision only. The approach of the rat to the response box turned on two LEDs inside the opening of the box, at which point the rat initiated visual sampling of the object stimulus. The rat then had to either push the object-attached response box to obtain a food reward from the food well underneath the response box (i.e., push response) or rear up and put its muzzle into the upper food well of the response box to obtain the food reward inside (i.e., nose-poke response), based on the identity of the object presented ahead. In scene trials, one of the scene stimuli was displayed via the three LCD monitors that surrounded the linear track when the rat activated the same optical sensor in front of the response box (Fig. 1*A*,*B*). During scene trials, no object stimulus was attached to the response box and LEDs were not turned on. Similar to the case for object trials, each scene stimulus was paired with only one of the behavioral responses. The scene stimulus disappeared when the animal made a response. Object and scene stimuli were presented in an intermixed fashion within the same session, with the restriction that the same stimulus did not appear in more than three consecutive trials. When rats reached the criterion of >75% performance on 2 consecutive days, the hyperdrive was implanted for electrophysiological recording.

### Hyperdrive implantation

Four fine nichrome wires (diameter, 17.8 μm) were wound together and heat bonded to form a tetrode. The wires were gold plated using a Nano-Z (Neuralynx) to lower the impedance to ∼200 kΩ at 1 kHz. For simultaneous recording from the PER and POR, a bundle of 27 tetrodes was housed in an elliptical 12G stainless steel cannula (anterior–posterior axis, 3.3–3.4 mm; mediolateral axis, 2.2–2.7 mm) and chronically implanted in the right hemisphere. Three of the 27 tetrodes were used only as reference electrodes and therefore could not record isolated single-unit activities. The temporalis muscle on the right side was fully retracted to position the bundle as laterally as possible. The electrode bundle was centered at the coordinates, 6.8 mm posterior to bregma and 4.5–5.8 mm from the midline. The bundle tip was angled laterally at 10° to 15°. We implanted the hyperdrive to target the PER and POR in eight rats, but because of the difficulty in targeting the slim band of rhinal cortices, two of the rats ended up with their tetrodes targeting only the intermediate hippocampus. We excluded those two animals, and all analyses on behavioral and neural data were conducted by using the remaining six rats. Among six rats, cells were recorded from both PER and POR in four rats and only from PER in two rats.

### Electrophysiological recording

During recovery from surgery, rats were acclimated to a custom-made sleeping booth outside the experimental room for 1 week. Food was restricted to reduce body weight to a presurgical level, and rats were retrained in the visual recognition task. The individual electrodes were lowered daily until the majority reached the upper border of the PER or POR; daily tip locations were recorded. No attempts were made to record the same neuron across days. Neural signals were differentially amplified 1000–10,000-fold and bandpass filtered (300–6000 Hz) using a Digital Lynx data acquisition system (Neuralynx). Spike waveforms exceeding a preset threshold (50–100 μV) were timestamped, digitized at 32 kHz, and stored for subsequent offline analyses.

### Histologic verification of recording sites

After all recording sessions were completed, the tip position was marked by making an electrolytic lesion with the small amount of current (10 μA current for 10 s) via each tetrode. After 24 h, the rats were killed with an overdose of CO_2_, then perfused transcardially first with PBS and then with a 4% (v/v) formaldehyde solution. After extraction, the brain was kept in a 4% v/v formaldehyde-30% sucrose solution at 4°C until it sank to the bottom of the container. The brain was subsequently coated with gelatin, soaked again in 4% (v/v) formaldehyde-30% sucrose solution, and then sectioned at a thickness of 40 μm using a freezing microtome (HM 430, Thermo Fisher Scientific). Each brain section was mounted and stained with thionin (for Nissl staining) or gold solution (for myelin staining). Photomicrographs of each brain section were obtained using a microscope mounted with a digital camera (model Eclipse 80i, Nikon). The exact tip positions of each tetrode were verified by comparing 3D reconstructed images with physiological depth profiles from each recording day. The boundary between the PER and POR was demarcated at the caudal limit of the angular bundle (−7.5 mm from bregma, based on the rat atlas; see Fig. 3*A*). Dorsal and ventral boundaries also followed the criteria of Burwell (2001; see Fig. 3*B*). As mentioned above, two of the rats did not have tetrode tips in the PER or POR and were eliminated from further analyses.

### Unit isolation

Single units were isolated using commercial software (SpikeSort 3D, Neuralynx). Various waveform parameters, including peak amplitude, energy, and peak-to-trough latencies, were used to distinguish single units from the PER (*n* = 66) and the POR (*n* = 51). Only units with mean firing rates ≥0.5 Hz during the event period (from cue onset to response) were analyzed.

### Single-unit analysis

#### Basic firing properties

Single units from the PER and POR were grouped into (1) bursting, (2) regular-spiking, and (3) unclassified neurons based on their autocorrelograms and interspike interval histograms (Barthó et al., 2004). Specifically, cells were classified as bursting neurons when they met the following criterion calculated from the counts in their autocorrelograms (Fig. 4):

max (3–5 ms)> max (0–50 ms)/2.

Among the remaining neurons, those in which the mode of the interspike interval histogram was <35 ms were classified as regular-spiking neurons. Unclassified neurons were those that showed neither bursting nor regular-spiking behavior.

Spike width was measured as the distance from peak to trough. Burst index was calculated according to the following formula from the counts in their autocorrelograms (Fig. 4) (Barthó et al., 2004):

$$\text{Burst index}=\frac{\text{max(3–5 ms)}}{\text{max(0–50 ms)}}.$$

#### Time normalization

Our behavioral paradigm allowed rats to voluntarily trigger the onset of a cue and make a choice response (i.e., push or nose-poke) in each trial. This led to variability in trial-by-trial latencies. To overcome the variability, we normalized the time of the event period (i.e., from cue onset to choice) for each trial to have an equal number of time bins (*n* = 30, average bin size = 57.46 ms). These normalized time bins were used to calculate firing rates in our further analysis (see below for details).

#### Generalized linear model

All analyses described below were performed on correct trials only. A Poisson generalized linear model (GLM) was fitted to the spiking data of each neuron with four binomial predictors using the “stepwiseglm” function in MATLAB. Each predictor represented the presentation of one of four stimuli (Tsao et al., 2018). A predictor (i.e., owl predictor) had a value of 1 in trials where the matched stimulus (i.e., owl object) was presented, whereas it had a value of 0 in other trials (i.e., for zebra, pebbles, or phone; see Fig. 6A). The stepwise selection of significant predictors was based on the *p*-value for an *F* test of the change of the sum of the squared error. We used *p* < 0.05 as the criterion for adding a predictor term and *p* > 0.1 for removing a predictor term from a GLM. The firing rates of each normalized time bin were fitted to a GLM. We categorized each neuron based on which predictor was included as a significant predictor in a GLM. If a GLM fitted to the neuron had only one significant predictor, the neuron was labeled as a “stimulus-selective cell” that selectively responded to a specific stimulus. We further divided stimulus-selective neurons into “object-selective” or “scene-selective” neurons based on the stimulus type (i.e., object or scene) of the significant predictor. Among neurons fitted to two significant predictors, we observed “response-selective” neurons that selectively fired to two stimuli that were associated with the same choice behavior (i.e., push or nose-poke). Response-selective neurons always had two significant predictors with the same sign (plus or minus) of coefficient in GLM, which reflected the consistent increase or decrease of the firing rate to a specific choice behavior. The remaining cells, namely those with no significant predictor or with two significant predictors that were not associated with the same choice response, were classified as others. No cell was fitted to three or four significant predictors.

#### Selectivity index

We calculated a “selectivity index” (SI) to quantify the strength of selective firing under a specific trial condition (i.e., stimulus or choice behavior) by calculating Cohen’s *d* for each time bin, as follows:

$$\text{Cohen’s }d\;=\text{ ±}\frac{\text{mean(FP) – mean(FNP)}}{\text{SD(FP,FNP)}},$$

where FP is the mean firing rate to the preferred stimulus and FNP is the mean firing rate to the other nonpreferred stimulus (see Fig. 7*A*,*C*). SI was obtained as the sum of Cohen’s *d* for all time bins. In calculating SI, the sign of Cohen's d was determined by whether a neuron showed higher firing rates (i.e., responsive, see Fig. 5*C*) or lower firing rates (i.e., nonresponsive, see Fig. 5*D*) to a preferred stimulus (see Table 2). Responsive neurons were assigned a positive Cohen’s *d* when their firing rates for the preferred stimulus exceeded those for nonpreferred stimuli (FP > FNP), and vice versa for inhibitory nonresponsive (FP < FNP), resulting in positive SI for all selective neurons. To compare the SI between real and shuffled data, we permutated trial identities within a recording session and calculated the SI as described above but with shuffled trial conditions (see Fig. 7*B*,*D*). We repeated the procedure 1000 times and averaged all 1000 values to get a single sum of SI from shuffling.

#### Data availability

The datasets generated and/or analyzed during the current study are available from the corresponding author on reasonable request.

## Results

### Basic firing properties of single units recorded during the VSOM task are generally similar between the PER and POR

In the VSOM task, rats (*n* = 6) were trained to associate four visual stimuli with the following two choice responses: two toy objects (phone and owl) and two visual patterns (i.e., scenes; zebra stripe and pebble patterns) were associated with either a “nose-poking” response to a food well located on top of a 3D-printed response box or a “pushing” response to the response box to retrieve a reward in the food well underneath it (Fig. 1*A*, Movie 1). In an object-cued trial, either the phone or owl object was presented as a cue inside the transparent show window in the front panel of the response box (Fig. 1*B*,*C*). The approach of a rat to the box activated LEDs installed inside the show window to illuminate the object cue, allowing the animal to visually sample the object. The owl and phone cues were associated with push and nose-poke responses, respectively (Fig. 1*A*). For a scene-cued trial, no object was displayed in the show window; instead, a visual scene was displayed on three adjacent LCD monitors that surrounded the apparatus (Fig. 1*C*). The zebra-stripe and pebble scenes were associated with push and nose-poke responses, respectively (Fig. 1*A*). To analyze the neural data in relation to the major events of the task, we defined an “event period” as the time from cue onset (i.e., the activation of object-illuminating LED in object-cued trials and the activation of visual scenes in scene-cued trials) to the choice response (push or nose-poke; Fig. 1*A*). The stimulus–response contingencies were counterbalanced between subjects. In a given session, object- and scene-cued trials were presented in a pseudorandom fashion.

Movie 1. The VSOM task. The video was recorded in a session where zebra scene and owl object were associated with nose-poke response, and pebble scene and phone object were associated with push response. 10.1523/ENEURO.0065-22.2022.video.1

**Figure 2.**
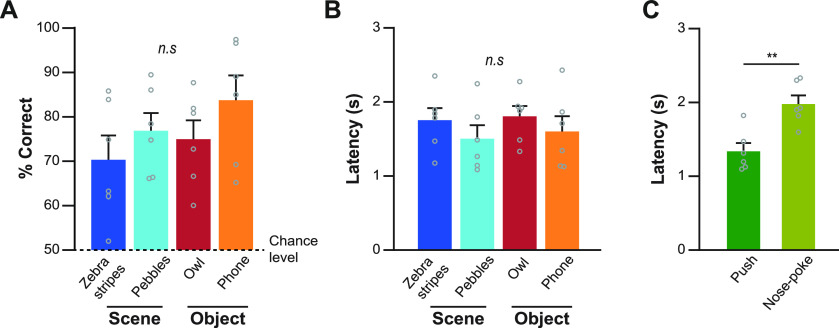
Behavioral performance in the VSOM task. ***A***, Behavioral performance (percent correct) was measured separately for each stimulus condition. Performance exceeded the chance level (50%) for all stimuli. No significant performance difference was observed between stimulus conditions. ***B***, Latency measured within the event period was plotted for each stimulus. There was no significant difference in latency. ***C***, Latency was compared between the two choice responses. The nose-poke response required a significantly longer latency compared with the push response. Each dot represents the average of a rat; data are presented as the mean ± standard error of the mean (SEM). **p < 0.01. n.s., Not significant.

To record single units in the VSOM task, each rat was used for three to seven sessions (mean = 4.67, SD = 1.37). The average performance levels of rats were significantly above chance for all stimuli (zebra: *t*_(5)_ = 3.69, *p* = 0.007; pebbles: *t*_(5)_ = 6.73, *p* = 0.0005; owl: *t*_(5)_ = 5.9, *p* = 0.001; phone: *t*_(5)_ = 5.99, *p* = 0.0009; one-tailed one-sample *t* test), whereas no significant difference was found in performance between stimuli (*F*_(3,15)_ = 1.75, *p* = 0.2, repeated-measures ANOVA; Fig. 2*A*). Response latencies (from cue onset to choice response) did not significantly differ among the stimuli (*F*_(3,15)_ = 0.68, *p* = 0.58, repeated-measures ANOVA; Fig. 2*B*). The latencies for the push and nose-poke responses were significantly different from each other (*t*_(5)_ = 5.2, *p* = 0.003, paired *t* test; Fig. 2*C*). This largely reflected differences in the required motor behaviors (e.g., pushing the response box was a quicker motion than standing up and nose poking in the food well) and did not lead to any difference in performance (push: mean = 76.2%, SD = 7.6%; nose-poke: mean = 78.6%, SD = 10.5%; *t*_(5)_ = 0.65, *p* = 0.54; paired *t* test).

**Figure 3.**
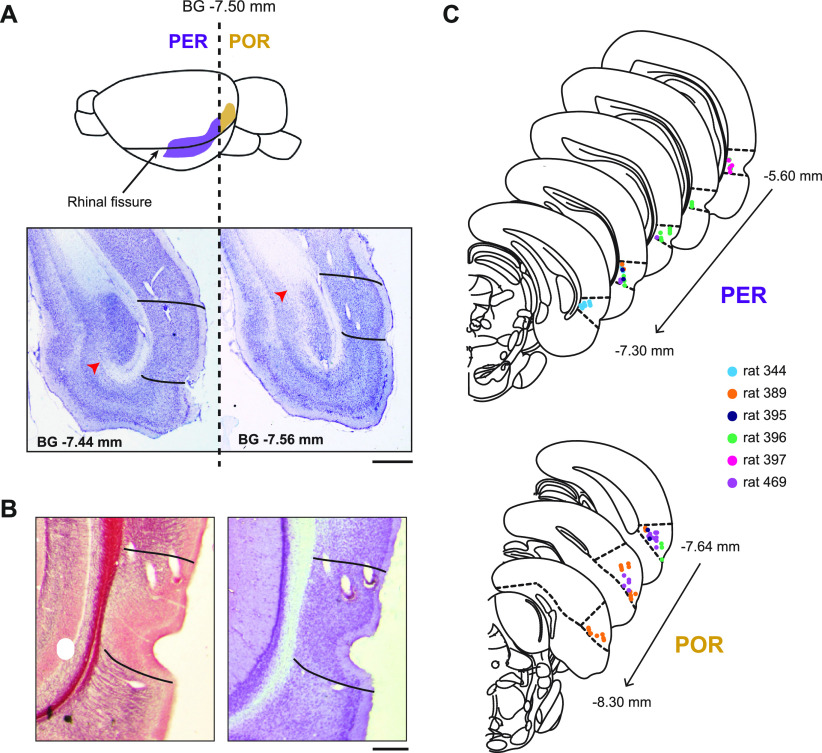
Histologic verification of recording sites in the PER and POR. ***A***, Lateral view of the rat brain (top). The PER and POR lie alongside the anterior and posterior strip of the rhinal fissure (marked by arrow). The vertical dash demarcates the boundary (−7.5 mm from bregma) between the PER (purple) and POR (gold). Thionin-stained sections near the boundary between the PER and POR (bottom). The caudal limit of the angular bundle (marked with red arrow) was defined as the border between the PER and POR (Burwell, 2001), which corresponds to −7.5 mm from bregma according to Paxinos and Watson (2009). Lines demarcate the boundaries of the PER or POR. Scale bar, 1 mm. ***B***, Dorsal and ventral borders of the PER and POR were defined based on adjacent myelin-stained (left) and thionin-stained (right) sections. Lines demarcate the boundaries of the PER. Scale bar, 0.5 mm. ***C***, Locations of recording electrodes in the PER and POR are marked in the nearest sections found in the atlas by Paxinos and Watson (2009). Different colors are used to mark electrodes from different rats.

**Figure 4.**
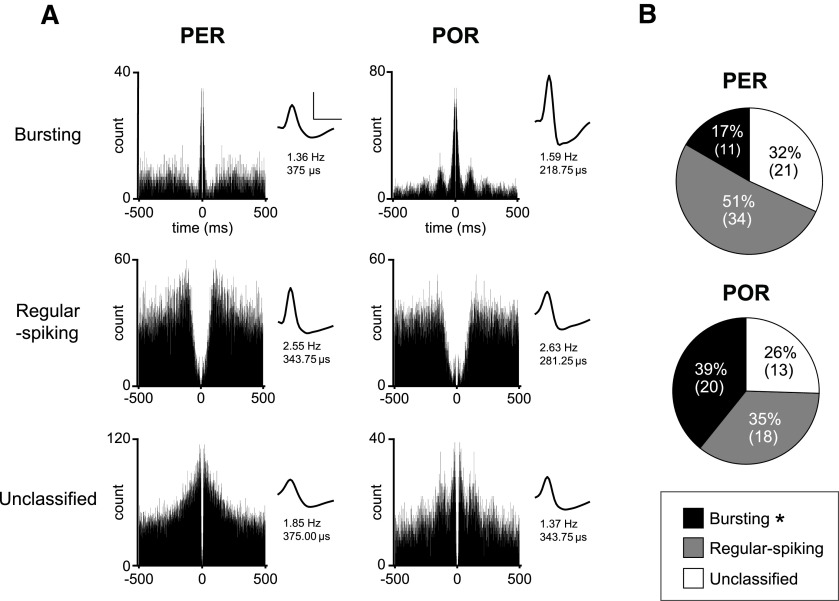
Basic firing properties of neurons in the PER and POR. ***A***, Representative autocorrelograms and waveforms of neurons from the PER and POR. The mean firing rate and spike width of a neuron are indicated below the waveform. Neurons were classified as bursting (top), regular spiking (middle), and unclassified (bottom) based on the study by Barthó et al. (2004). Calibration: amplitude (vertical bar), 100 μV; width (horizontal bar), 500 μs. ***B***, Pie charts showing the proportions of three neuronal categories for the PER (top) and POR (bottom). Regular-spiking neurons were more abundant in the PER, whereas more bursting neurons were present in the POR. The numbers in the parentheses denote the number of neurons. *p < 0.05.

We recorded the spiking activities of single units in the PER and POR using a hyperdrive housing 24 tetrodes to target the areas along the anteroposterior axis of the rhinal fissure (Fig. 3*A*). The boundaries between the PER and POR were demarcated by the caudal limit of the angular bundle, which was −7.5 mm from bregma based on the atlas (Fig. 3*A*, red arrowheads; Burwell, 2001). Tissue sections subjected to myelin and thionin staining were used to determine the dorsal and ventral borders of the PER and POR (Fig. 3*B*). Single units in the PER (*n* = 66; 5.20–7.44 mm posterior to bregma) and POR (*n* = 51; 7.56–8.64 mm posterior to bregma) were recorded (Fig. 3*C*). Using the autocorrelogram as the major criterion, cells were classified as “bursting,” “regular-spiking,” and “unclassified” neurons based on their spiking properties (Barthó et al., 2004; Fig. 4*A*). Bursting cells were those with a large peak at 3–6 ms with an exponential decay after the peak. Regular-spiking cells showed an exponential rise of up to tens of milliseconds with a peak at <35 ms. Cells showing other patterns were labeled as unclassified. Comparison of the proportions of these neuron types between the PER and POR revealed that there was a significant regional difference (*p* = 0.024; Fisher’s exact test). A *post hoc* analysis showed that bursting cells were more frequently observed in the POR than in the PER (*p* = 0.01; Fisher's exact test), whereas the proportions between the two regions were similar for the other types of neurons (regular spiking, *p* = 0.09; unclassified, *p* = 0.54; Fisher’s exact test; Fig. 4*B*).

Mean firing rate, spike width, and burst index were compared between the two regions and among different cell types. Mean and SD values are presented in Table 1. There was no significant effect of region or cell type on the mean firing rate (region: *F*_(1,116)_ = 0.12, *p* = 0.73; cell type: *F*_(2,116)_ = 0.66, *p* = 0.52; two-way ANOVA) or spike width (region: *F*_(1,116)_ = 0.59, *p* = 0.45; cell type: *F*_(2,116)_ = 2.86, *p* = 0.06; two-way ANOVA). There was also no significant effect of region × cell type interaction on either the mean firing rate (*F*_(2,116)_ = 1.74, *p* = 0.18) or the spike width (*F*_(2,116)_ = 2.93, *p* = 0.058, two-way ANOVA). The bursting levels of bursting cells were not significantly different between the regions (*t*_(29)_ = 1.37, *p* = 0.18; unpaired *t* test). In general, we found no significant difference in the basic firing properties, such as the mean firing rate (*t*_(115)_ = 0.31, *p* = 0.76) or spike width (*t*_(115)_ = 1.34, *p* = 0.18) between the PER and POR in the current study.

**Table 1:** Mean firing rate, spike width, and burst index of PER and POR neurons

Brain region	Category	Mean firing rate (Hz)	Spike width (ms)	Burst index
PER	Regular spiking	3.3 (4.5)	312 (67)	
	Bursting	2.6 (2.4)	341 (26)	0.74 (0.25)
	Unclassified	4.9 (7.3)	296 (43)	
	All	3.7 (5.3)	311 (56)	
POR	Regular spiking	1.7 (1.5)	351 (69)	
	Bursting	5.4 (10.2)	316 (46)	0.61 (0.26)
	Unclassified	2.4 (2)	308 (54)	
	All	3.4 (6.6)	326 (59)	

Basic firing properties of neurons in the PER and POR were compared. The average values of mean firing rate (Hz), spike width (μs), and burst index of bursting neurons are presented according to cell types. The values in the parentheses are the SDs.

### Neurons coding choice responses, but not stimulus type, are more prevalent in the POR than the PER

**Figure 5.**
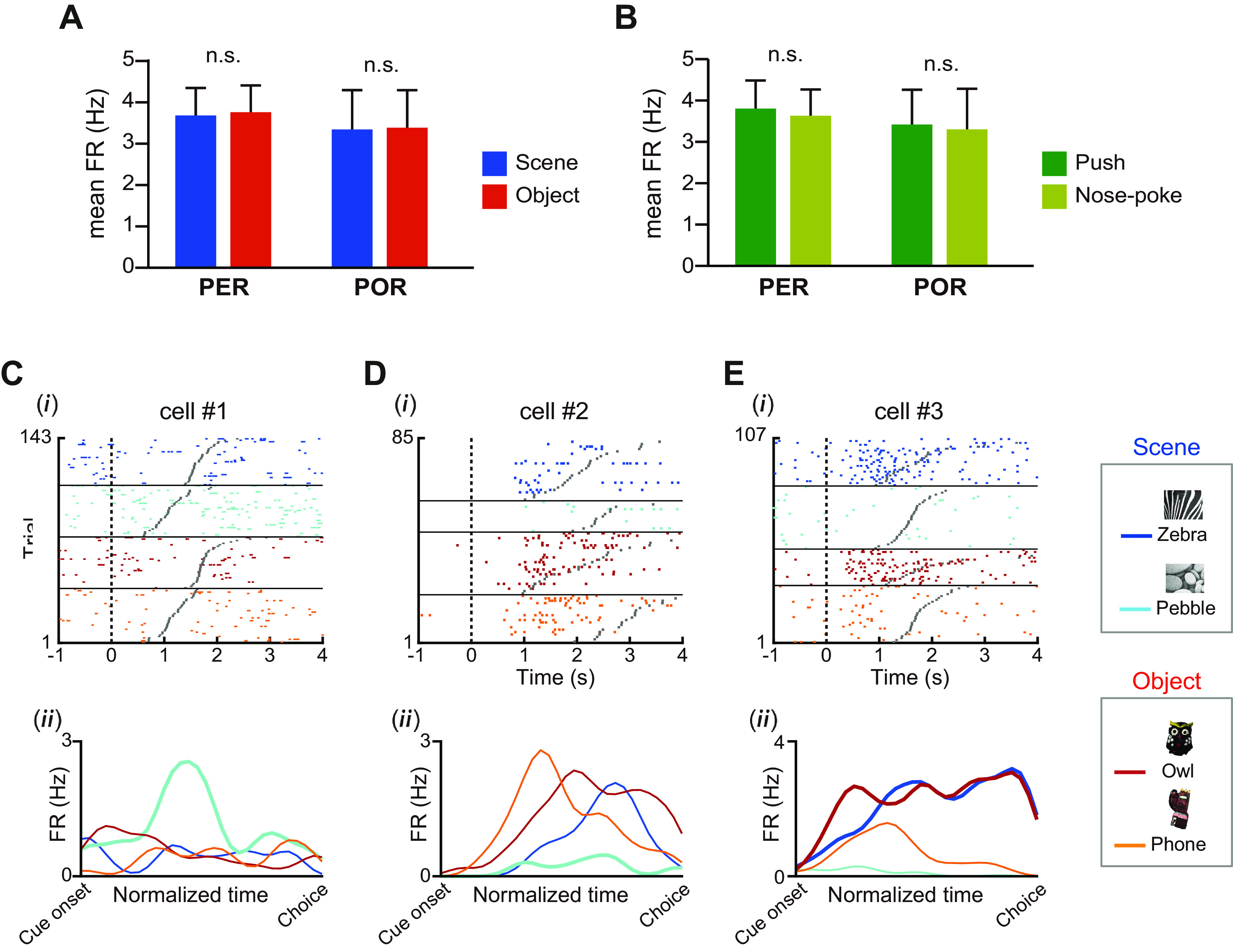
Characteristic firing patterns observed under various task-related conditions in PER and POR neurons. ***A***, Firing rates (FR) under different stimulus types (scene or object) were measured within the event period and compared between the PER and POR. Despite the difference in visual salience, scene and object stimuli evoked similar firing rates in the PER and POR. ***B***, Firing rates for different response conditions (push or nose-poke) were compared between PER and POR. No significant difference was observed in firing rates under different choice responses. Data are presented as the mean ± SEM. n.s., Not significant. ***C***, Firing patterns were examined by plotting the raster plot (***i***) and spike density functions (***ii***). In the raster plot, trials were grouped into four stimulus conditions and sorted based on the trial latency of the event period (i.e., from the onset of a stimulus marked with a dotted line to the choice response marked with a gray dot). Spike density functions for each stimulus condition were constructed within the event period based on normalized time bins. Cell 1 preferentially fired to the pebble scene (thick line), while it remained silent to the other stimuli (thin lines). ***D***, The raster plot (***i***) and spike density functions (***ii***) of cell 2, which also showed preferential firing patterns for the pebble scene (thick line), but by remaining silent compared with those observed for the other stimuli (thin lines). ***E***, The raster plot (***i***) and spike density functions (***ii***) of cell 3 firing for the zebra and owl stimuli (thick lines), which were associated with the same choice response (i.e., nose-poke).

We first examined whether there were differences in firing rates between the PER and POR according to the stimulus type (i.e., object and scene). Although one may think that the visual scene stimuli could be visually more salient to rats than the object stimuli in our experimental setup, the mean firing rates in the event period were not significantly different between the two regions (*F*_(1,115)_ = 0.1, *p* = 0.75) or stimulus types (*F*_(1,115)_ = 1.32, *p* = 0.25, two-way repeated-measures ANOVA; Fig. 5*A*). No significant interaction between region and stimulus type was found (*F*_(1,115)_ = 0.12, *p* = 0.73). We examined the differences in firing rates for the push and nose-poke trials, but did not find any significant main effect of region (*F*_(1,115)_ = 0.11, *p* = 0.74) or response (*F*_(1,115)_ = 0.97, *p* = 0.33, two-way repeated-measures ANOVA; Fig. 5*B*). There was also no significant interaction of region and response factor (*F*_(1,115)_ = 0.04, *p* = 0.85). These findings suggest that efforts to differentiate the PER and POR with respect to their roles in the VSOM task may require more sophisticated measures of neuronal activity than measurement of the average firing rate.

To identify task-related firing correlates, we constructed a raster plot using all correct trials from each stimulus condition in each recording session (Fig. 5*Ci*,*Di*,*Ei*). Since the latencies of individual event periods (from the dotted line to the gray dot in raster plots) varied among trials (Fig. 2*C*), we normalized the latency of each trial such that every trial contained the same number of time bins (*n* = 30). By plotting the spike density function based on the normalized time bins, we observed three characteristic firing patterns in neurons recorded from both regions (Fig. 5*Cii*,*Dii*,*Eii*). Some neurons fired preferentially for a specific stimulus by increasing their firing rates compared with those seen for the nonpreferential stimuli (Fig. 5*C*), whereas other neurons showed low firing rates (or remained silent) to indicate such stimulus preference (Fig. 5*D*). For example, cell 1 selectively increased its firing rate within the event period during which the pebble scene was presented (Fig. 5*C*). In contrast, cell 2 exhibited its preference for the pebble scene by remaining silent only under that stimulus (Fig. 5*D*). The third class of neurons showed preferential firing to two stimuli associated with the same choice response, and thus may code a response instead of a specific stimulus. An example of this is cell 3, which consistently fired in trials in which the rat nose-poked in response to the zebra and owl stimuli (Fig. 5*E*).

**Figure 6.**
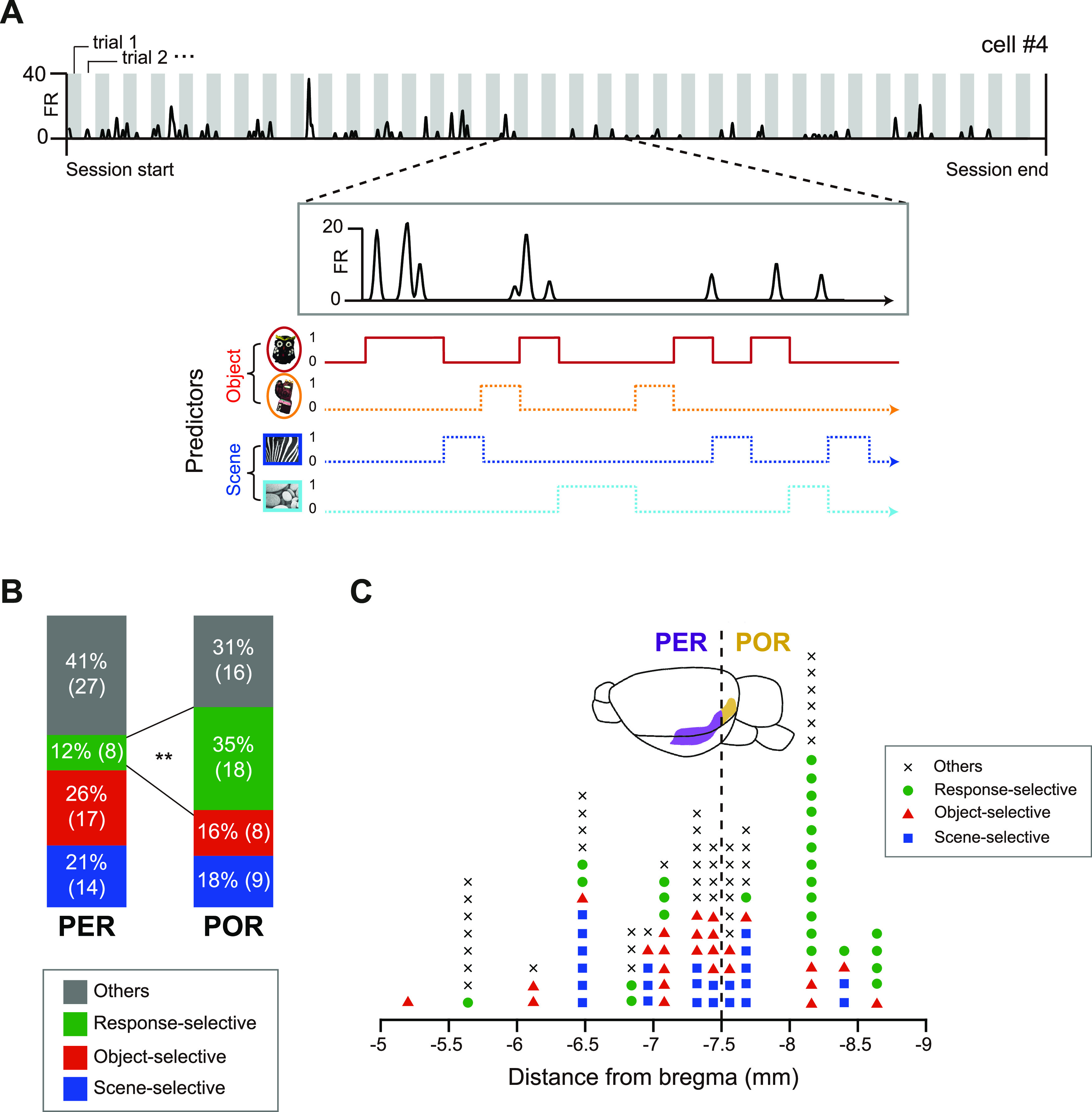
The PER and POR differ in their proportions of response-selective neurons but not stimulus-selective neurons. ***A***, An analytic scheme showing the cell-categorization process. The firing rates of an example neuron from a session were fitted to a GLM using four predictors (or stimuli). In the extended plot of a gray box, the neuron increased its firing rates in trials where the owl object was presented (i.e., trials where the owl predictor had a value of 1). The GLM for the example neuron selected the owl predictor as a significant predictor (solid line), while the other predictors were found to be unsuitable for predicting the firing rates (dotted lines). ***B***, Proportions of cell categories determined by GLM analysis. We categorized neurons based on which predictor was included in a GLM explaining the firing rates of the neuron. Stimulus-selective neurons were those with only one significant predictor in a GLM, implying preferential firing to a specific stimulus. Among stimulus-selective neurons, we further dissociated object- and scene-selective neurons based on which type of predictor (object or scene) was significant. Response-selective neurons were those with two significant predictors that were associated with the same choice response (push or nose-poke). Statistical comparison revealed that there were higher proportions of response-selective neurons in the POR compared with the PER. **p < 0.01. ***C***, Functional categories of all recorded neurons and their anatomic locations. The dotted line represents the border between the PER and POR. Response-selective neurons (marked with green circles) were abundant in relatively posterior regions of the postrhinal cortex. We did not observe any clear functional segregation within regions neighboring the border.

To objectively categorize neurons based on their preference for task-related conditions, we used a stepwise GLM to avoid multiple comparisons among conditions (Tsao et al., 2018). For this purpose, a Poisson GLM was fitted to each neuron based on the firing rates in the normalized time bins within the event period, and each stimulus was used as a predictor for GLM fitting (Fig. 6*A*). For example, when the owl object was presented as a cue in a trial, the trial was given a value of 1 for the owl predictor; when the other predictors were presented, the trial was given a value of 0 for the owl predictor. For instance, cell 4 in Figure 6*A* preferentially increased its firing rate when the owl object was presented and was relatively silent for the other stimuli. The GLM for this example neuron therefore identified only the owl predictor as a significant predictor (Fig. 6*A*).

We classified neurons based on which predictors were selected as significant predictors in the GLM. A neuron found to be associated with a single significant predictor was considered to be a “stimulus-selective” neuron. The stimulus-selective cells were further divided into scene-selective or object-selective neurons based on the type of the stimulus (i.e., scene or object) found to be a significant predictor. For example, since cell 4 had the owl predictor as a significant predictor in the GLM, the neuron was classified as being an object-selective neuron (Fig. 6*A*). We defined response-selective neurons (Fig. 5*E*, cell 3) as neurons having two significant predictors (or stimuli) that were associated with the same choice response in the GLM. Neurons that did not meet the above criteria (i.e., those with no significant predictor or for which two significant predictors were associated with different responses) were classified as “others.” No neuron showed more than three significant predictors.

Based on the procedures described above, we compared the proportions of cells of the functional categories and found that there was a significant difference between the PER and POR (*p* = 0.003, Fisher’s exact test; Fig. 6*B*). For the stimulus type, we did not observe any significant difference between the two regions in terms of the proportion of cells coding scenes (*p* = 0.41) or objects (*p* = 0.14; Fisher's exact test). However, there was a significantly higher proportion of neurons coding specific choice responses in the POR compared with the PER (*p* = 0.003, Fisher's exact test with Bonferroni correction). Stimulus-selective neurons showed preferential firing patterns either by higher firing rates to the preferred stimulus (Fig. 5*C*) or by lower firing rates to the preferred one (Fig. 5*D*). We defined the formal type as “responsive” and the latter type as “nonresponsive,” and they were determined by the sign of the β-coefficient in the GLM (i.e., the plus sign in responsive types and the minus sign in nonresponsive types). Both responsive and nonresponsive types of neurons for objects and scenes were found in the PER and POR (Table 2). For response-selective neurons, we did not differentiate responsive and nonresponsive types because they were always responsive to two stimulus conditions, and nonresponsive to the other two conditions regardless of the sign of the β-coefficient of the GLM. Together, our findings suggest that the most salient functional dissociation between the PER and POR arises from a difference in the proportion of response-selective neurons.

**Table 2:** Responsive or nonresponsive type of stimulus-selective neurons in the PER and POR

Brain region	Coding stimulus type	Responsive (plus sign in GLM)	Nonresponsive (minus sign in GLM)
PER	Object	11 (65%)	6 (35%)
	Scene	7 (50%)	7 (50%)
POR	Object	6 (75%)	2 (25%)
	Scene	4 (44%)	5 (56%)

Stimulus-selective neurons showed either the responsive or nonresponsive type of firing for a specific stimulus. The type was determined by the sign of β-coefficient in the GLM of each neuron. Stimulus-selective neurons with plus signs are responsive (i.e., high firing rates to a preferred stimulus), and the ones with minus signs are nonresponsive (i.e., low firing rates to a preferred stimulus) types.

Since the PER and POR are adjacent to each other along the anterior–posterior axis (Fig. 3*A*), our result might have been confounded by spiking activities recorded from the tetrodes whose recording positions were located near the boundaries between the PER and POR. To test this possibility, we examined the distances of the tetrodes relative to bregma and marked the functional categories of the neurons recorded from those tetrodes (Fig. 6*C*). From >40 neurons recorded near 7.5 mm posterior to bregma, we did not observe any clear categorical bias. Neurons coding a choice response were abundant in the more posterior part of the rhinal cortical area (>8 mm posterior to bregma). Together, these results suggest that the higher proportion of response-selective neurons we found in the POR was not mainly attributable to a sampling bias near the PER–POR borders.

### Response-selective neurons fire more selectively for different responses than the stimulus-selective neurons do for different cue stimuli in the PER and POR

**Figure 7.**
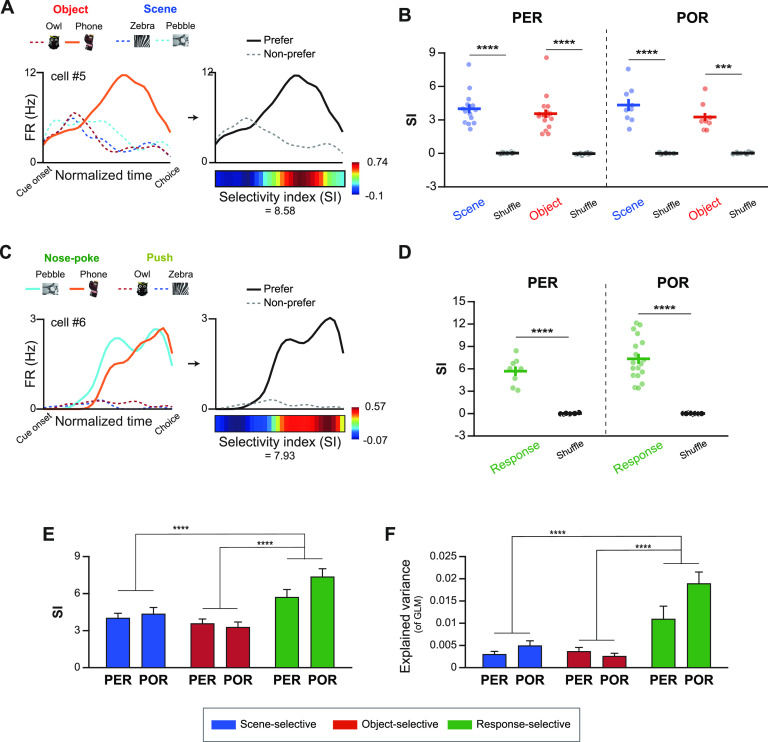
Quantification of selective firing patterns from stimulus- and response-selective cells. ***A***, An example of quantifying selective firing patterns in stimulus-selective neurons. The neuron classified as an object-selective neuron preferentially fired to the phone object (orange solid line). Firing rates for the phone object were designated as Prefer (black solid line), while firing rates for the other stimuli were averaged to be Non-prefer (gray dotted line) firing rates. To define the SI, Cohen’s *d* was calculated between the firing rates to Prefer and Non-prefer in each time bin and plotted as a heatmap. The SI was taken as the sum of Cohen’s *d* for all time bins. ***B***, The SI was compared with shuffled data to verify that cell categorization based on GLM matched with averaged firing patterns. Scene- and object-selective neurons in both PER and POR showed higher SI compared with the shuffled data. ***C***, An example of a response-selective neuron and its SI. The example neuron increased its firing in nose-poke trials. Firing rates for the nose-poke condition were averaged to Prefer firing rates, and for the push trials were averaged to Non-prefer firing rates. SI was calculated following the same procedure. ***D***, Response-selective neurons also showed higher SI compared with shuffled data in the PER and POR. ***E***, The SI was compared between regions and categories. Response-selective neurons of the PER and POR showed significantly higher SI compared with scene- and object-selective neurons, regardless of region. ***F***, The explained variance (adjusted *R*^2^) obtained from the GLM generated in the previous analysis was compared between regions and categories. The GLM had a significantly higher explained variance for response-selective neurons compared with the other categories. Data are presented as the mean ± SEM. ***p < 0.001, ****p < 0.0001.

We quantified the strength of selectivity of each neuron for a stimulus type or response based on its averaged firing patterns to (1) verify whether our GLM-based classification properly reflected the firing patterns of each neuron and (2) compare the strength of neuronal selectivity between regions and task-related conditions. We calculated an SI, which was defined as the sum of Cohen’s *d* between the firing rates to the preferred stimulus (or stimuli) and the firing rates to the nonpreferred stimuli. For example, cell 5 had the phone object as a significant predictor in its GLM (Fig. 7*A*, left). Therefore, the firing rates associated with the phone object were designated as “Prefer” and those for the other three stimuli were averaged and designated as “Non-prefer” (Fig. 7*A*, right). Cohen’s *d* was calculated for each normalized time bin, and the sum of all time bins was taken as the SI for that neuron.

We examined whether the stimulus-selective neurons did, indeed, exhibit preferential firing patterns for a particular stimulus condition by comparing SI values with those obtained for shuffled datasets in which the trial identities were permuted. The SI calculated from the actual data were significantly higher than that for the shuffled data for both the PER (scene: *t*_(13)_ = 9.85, *p* < 0.0001; object: *t*_(16)_ = 9.26, *p* < 0.0001) and the POR (scene: *t*_(8)_ = 7.9, *p* < 0.0001; object: *t*_(7)_ = 7.27, *p* = 0.0002; paired *t* test; Fig. 7*B*). For response-selective neurons (Fig. 7*C*, cell 6), the firing rates for the two stimuli (i.e., those associated with the nose-poke response) identified as significant predictors in the GLM were averaged as Prefer and the other two spike density functions (i.e., those for push responses) were averaged as Non-prefer (Fig. 7*C*). We calculated the SI for the response-selective neurons using the same procedures described for the stimulus-selective neurons. Response-selective neurons also showed significantly higher SI compared with the shuffled data for both the PER (*t*_(7)_ = 9.03, *p* < 0.0001) and the POR (*t*_(17)_ = 11, *p* < 0.0001; paired *t* test; Fig. 7*D*).

When the SI was compared between categories and regions, we found that the response-selective signals were always stronger than the stimulus-selective signals in both regions (Fig. 7*E*). Two-way ANOVA of the SI revealed a significant main effect for the functional category (*F*_(2,68)_ = 15, *p* < 0.0001) but not the region (*F*_(1,68)_ = 1.36, *p* = 0.25). There was no significant interaction between category and region (*F*_(2,68)_ = 1.42, *p* = 0.25). *Post hoc* analysis showed that the SIs of the response-selective neurons were significantly higher than those of the scene-selective neurons (*t*_(47)_ = 4.3, *p* < 0.0001) or object-selective neurons (*t*_(49)_ = 5.6, *p* < 0.0001; unpaired *t* test with Bonferroni correction). This result was in line with the results obtained from two-way ANOVA comparing the explained variance (adjusted *R*^2^) of the GLM in the previous analysis (Fig. 7*F*). We discovered a significant main effect for the cell category (*F*_(2,68)_ = 21.11, *p* < 0.0001), but not for the region (*F*_(1,68)_ = 3.15, *p* = 0.08). The interaction between the cell category and region was not statistically significant (*F*_(2,68)_ = 2.59, *p* = 0.08). *Post hoc* analysis revealed that the adjusted *R*^2^ values for response-selective neurons were significantly higher than those for scene-selective neurons (*t*_(47)_ = 5.39, *p* < 0.0001) or object-selective neurons (*t*_(49)_ = 5.76, *p* < 0.0001; unpaired *t* test with Bonferroni correction). These results indicate that the differential signals for response-selective neurons were stronger than those for stimulus-selective neurons in both the PER and the POR.

### Stimulus and response factors differentially influence the neural firing patterns in the PER and POR

Above, we presented evidence that the PER and POR are dissociated by their proportions of response-selective neurons, and the response-selective neurons showed greater contrast in their selectivity compared with neurons coding specific stimuli. One may take these results as indicating that stimulus- or response-selective cells are modulated only by a single task-related variable: either stimulus or response information. However, it remained possible that neural firing patterns could be significantly modulated by both stimulus and response factors, but to different degrees. To address this, we investigated whether stimulus-selective (or response-selective) neurons were differentially modulated by the response factor (or by the stimulus factor) between the PER and POR.

**Figure 8.**
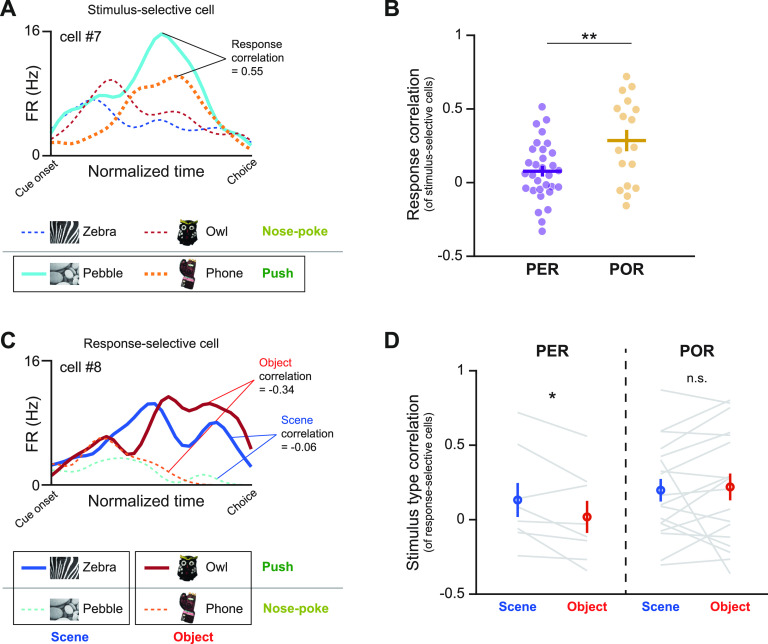
Further dissociation of PER and POR functions based on the interaction of stimulus type and response. ***A***, The response correlation of stimulus-selective cells was calculated. For example, the response correlation in cell 7 (selective to the pebble scene) was calculated between firing patterns for pebble and phone, both of which required the push response. ***B***, The response correlation of stimulus-selective neurons was compared between PER and POR. POR neurons had a significantly higher response correlation, indicating that stimulus-selective neurons in POR were more strongly influenced by the response factor. ***C***, Stimulus-type correlations for scene and object were calculated in response-selective cells. Cell 8, which preferentially fired for the push response, showed distinct firing patterns between the two object stimuli, as reflected by the negative correlation coefficients obtained within the object conditions. The correlation between scene conditions was relatively high. ***D***, Scene and object correlations from response-selective neurons were compared. For response-selective neurons of the PER, object correlation was significantly lower than scene correlation. Response-selective neurons in the POR were not modulated by stimulus type, maintaining a similar level of correlation between scene and object. Data are presented as the mean ± SEM. *p < 0.05, **p < 0.01.

For this purpose, we first examined how stimulus-selective neurons were modulated by choice response. We calculated the correlation of the firing patterns between the preferred stimulus and the stimulus that required the same choice response as the preferred one. For example, cell 7 from the POR in Figure 8*A* was classified as a stimulus-selective neuron by its preferential firing for the pebble scene. This neuron was recorded in a session where the pebble scene and phone object were associated with the push response. Although the firing rate associated with the phone object was relatively low, the firing patterns were highly correlated with the pebble scene condition. The Pearson’s correlation between the firing patterns for the pebble scene and the phone object was defined as the “response correlation” (of the stimulus-selective cell). We compared the obtained response correlations for the stimulus-selective cells between the PER and POR (Fig. 8*B*). Our results revealed that the response correlations were significantly higher in the POR than in the PER (*t*_(46)_ = 2.87, *p* = 0.006; unpaired *t* test), indicating that stimulus-selective neurons in the POR were more highly influenced by the response factor.

Next, we examined how response-selective neurons were modulated by different stimulus types. To quantify the bias of the response-selective neurons from the PER or POR toward processing (or dissociating) a specific stimulus type, we calculated a “stimulus-type correlation” (of response-selective neurons) for scene or object. The Pearson’s correlation between the firing patterns associated with the zebra and pebble scenes (or between those of the owl and phone objects) was defined as the “scene correlation” (or “object correlation”). For example, PER-localized cell 8 in Figure 8*C* was categorized as a response-selective neuron based on its preferential firing for the push response. When we divided the firing patterns according to scene and object conditions, the firing patterns between the two object conditions were highly dissociated, showing a negative object correlation. Scene correlation, on the other hand, was relatively high compared with object correlation in the same neuron (cell 8). When we compared the scene and object correlation within each region (Fig. 8*D*), we found that object correlation was significantly lower than scene correlation in the PER (*t*_(7)_ = 2.47, *p* = 0.04; paired *t* test). This finding may indicate that there is a functional bias toward object information processing in response-selective neurons of the PER. However, the response-selective neurons in the POR did not show a significant difference between the scene and object correlations (*t*_(17)_ = 0.37, *p* = 0.72; paired *t* test). These results collectively suggest that both stimulus and response types and, most importantly, their interactions need to be considered if we hope to capture the functional differentiation between the PER and POR.

## Discussion

In the current study, we recorded single neurons in the PER and POR from rats performing a recognition memory task involving two types of visual stimulus (object and scene). Rats were required to choose a pushing or nose-poking response to get a reward based on the identity of a pseudorandomly presented stimulus. Neurons in the PER and POR fired selectively for a specific stimulus or response. For the stimulus type, similar proportions of neurons were selectively active for a scene or object stimulus in both regions; for response, in contrast, more response-selective neurons were found in the POR than in the PER. In addition, response-selective neurons showed more differential firing than stimulus-selective neurons. Importantly, our results emphasize that the strong “response” component in neural firing should be considered together with the stimulus information to better understand the functional differentiation between the PER and POR. Although we only recorded neural activities in well trained animals and thus could not monitor the neural correlates of acquisition, this does not necessarily mean that our task was not a memory task. Successful performance in our task requires normal object recognition memory. Our previous study has reported the neural activity often taken as physiological evidence of object recognition (i.e., repetition suppression) in the same behavioral paradigm in well trained rats (Ahn et al., 2019).

Some prior studies tested the effects of lesioning or inactivating these regions using a similar behavioral paradigm (Bussey et al., 1999; Bucci et al., 2000; Myhrer, 2000; Norman and Eacott, 2005). A few studies also recorded the spiking activities of single units in the PER and POR in a spatial working-memory task on a plus maze (Burwell et al., 1998; Burwell and Hafeman, 2003), which focused on comparing spatial selectivity between the two regions. However, to our knowledge, this is the first study in which responses from single units were recorded from the PER and POR within the same subject in a single behavioral session while rats performed a recognition memory task using objects or scenes.

It has been reported that temporally inactivating the PER or POR resulted in performance deficits when a visual object or scene was used as a cue (Park et al., 2017), and our current findings may provide direct physiological evidence to support the prior results. In the previous study, real 3D objects placed behind a transparent acrylic barrier were used as visual object stimuli, and the same zebra stripes and pebble patterns were used as scene stimuli. Rats were required to associate the identity of each stimulus with the left or right choice in a T-maze, or digging or pushing a sand-filled jar. The response types used in the current study modified the previous digging or pushing responses (for a sand-filled jar) to make the apparatus more amenable to electrophysiological recording of single units during the behavioral task. We found that similar proportions of neurons selectively represented either object-type or scene-type stimuli. These results support the previous behavioral finding that there were comparable deficits in performance between visual object-cued and scene-cued trials on inactivation of the PER or POR (Park et al., 2017). This suggests that there is functional overlap between the PER and POR in object and scene information processing during a recognition memory task.

The traditional view has suggested that there are two parallel information-processing streams in the MTL. According to this view, the POR and its immediate downstream structure, the MEC, process spatial information, whereas the PER and its downstream structure, the LEC, are concerned mostly with nonspatial information, and the two thus form independent information-processing streams in the MTL before they merge in the hippocampus. The information-processing streams in this dual-stream theory are defined mostly by the type of sensory-perceptual information (i.e., spatial vs. nonspatial type) fed to the memory systems in the MTL (Knierim et al., 2014). Based on this content-based dual-stream model, an object is considered to generate nonspatial information, whereas a visual scene is frequently treated as spatial information, mainly because of its utility as an allocentric cue in spatial navigation (Epstein, 2005; Litman et al., 2009; Ranganath and Ritchey, 2012; Neunuebel et al., 2013; Maguire et al., 2016). According to this view, the object and scene information types are expected to be processed in the PER and POR, respectively.

However, the results from our studies overall suggest that the PER and POR may not always be dissociable based solely on the characteristics of sensory inputs. Indeed, there is a growing body of evidence now indicating that there are anatomic and functional overlaps between the so-called spatial and nonspatial information streams in the MTL (Burke et al., 2018). Doan et al. (2019) reported direct anatomic and physiological projections from the POR to the LEC. Furthermore, POR neurons mainly responded to the conjunction of an object and its spatial location, but not to a specific spatial location in the environment (Furtak et al., 2012). In addition, some PER neurons showed sustained activity to a large patch of the spatial layout (Bos et al., 2017) and fired in association with spatial locations in the absence of objects (Burke et al., 2012). These findings support the idea that the PER takes part in spatial information processing. The relatively unclear dissociation between the PER and POR may also be reflected in the seemingly related findings in their downstream regions. That is, in the MEC, object-vector cells that code a specific distance and direction information from an object have been reported (Høydal et al., 2019), and some LEC neurons could represent the egocentric bearings of the rat to the boundaries in an open arena even when there was no object cue (Wang et al., 2018). Our results further emphasize the need for revising the traditional dual-stream model to incorporate dynamic interplays of spatial and nonspatial coding at multiple levels in the MTL (Connor and Knierim, 2017; Burke et al., 2018; Doan et al., 2019; Fiorilli et al., 2021).

In our study, the choice responses more clearly differentiated the neural firing patterns between the PER and POR, compared with the stimulus types. In addition, a larger contrast in firing patterns was observed for the response factor than for the stimulus component (scene or object) in both regions. Prior studies suggested that the PER and POR are situated at the lowest level of the information-processing hierarchy in the MTL (Eichenbaum, 2000; van Strien et al., 2009). If this is correct, there should be a stronger feedforward influence for sensory information than response information. However, our findings suggest that this view may need to be revised to reflect more dynamic stimulus–response interactions at the level of the PER and POR. Furthermore, despite the anatomic implication that the POR may be more strongly influenced by visual inputs than the PER (Burwell and Amaral, 1998a), visually salient scenes failed to evoke stronger neural activity in the POR compared with the PER in our study. This observation highlights the need for a more complex model that can integrate both feedforward and feedback communications within the MTL. Notably, the PER and POR have reciprocal connections with the prefrontal cortex (Hwang et al., 2018) and the feedback signals from the prefrontal cortex may underlie the strong response-related coding in the PER and POR (Peng and Burwell, 2021).

Neurons coding a behavioral response were previously reported in the PER and POR when animals were required to produce an associated behavior based on a stimulus (Furtak et al., 2012; Ahn and Lee, 2015; Bos et al., 2017; Ohnuki et al., 2020). Those response-related firings were present even when the associated stimuli were from different sensory modalities (Ohnuki et al., 2020). Our previous study showed that the emergence of response coding was not confined to a time point at which animals were actually moving their muscles to produce a certain choice response (Ahn and Lee, 2015). Our present findings not only support these results, but also emphasize that response coding could be the key dissociating factor that controls the information flow in the MTL systems. It is still unclear what drives response coding in the PER and POR. Response coding in the MTL is likely to be made of heterogeneous components, including the motor behavior itself, the reward or value associated with the behavior, and the association with stimuli. Additional experiments are needed to dissociate the neural substrates that contribute to these components in connection with the PER and POR networks.

We herein found that response-selective neurons were more abundant in the POR than in the PER. As mentioned above, the mechanism underlying this phenomenon cannot be firmly ascertained by the current study, since the origin of response coding was not clear in these regions. One possibility is that the egocentric response coding associated with the push and nose-poke responses is represented more strongly in the POR than in the PER. This reasoning is based on the anatomic observation that the POR has connections with the retrosplenial cortex and posterior parietal cortex, both of which have been studied with respect to egocentric information processing (Burwell and Amaral, 1998a). Recordings from the POR also revealed that single neurons could represent the egocentric direction of an animal toward a specific location in two-dimensional space (LaChance et al., 2019). It is noteworthy that in passive-viewing paradigms, neurons in the POR were mostly responsive when a moving dot, rather than a fixed visual stimulus, was presented in rodents (Nishio et al., 2018). Together, these findings suggest that neurons in the POR may have responded to a push or nose-poke response in our study because rats experienced very different visual views, in egocentric terms, when making movements for pushing or nose-poking.

It is important to note that our experimental paradigm was designed to test animals in a goal-directed task, as opposed to experiments that measured only spontaneous behaviors, such as object exploration, foraging, and conditioned freezing (Bucci et al., 2000; Norman and Eacott, 2005; LaChance et al., 2019). Indeed, a previous study demonstrated that the stimulus, the task demand (i.e., behavioral response), and the interaction between them should be collectively considered when one seeks to understand the functional dissociations among different brain regions in a goal-directed situation (Lee et al., 2021). Specifically, the MEC is engaged when spatial navigation-related responses (e.g., left and right turns in a T-maze) are associated with visual scenes, whereas the LEC is required when non-navigational responses (e.g., pushing or digging a jar) are required (Yoo and Lee, 2017). Although the current study did not test different types of response profiles (i.e., navigational versus non-navigational), we herein show that neurons in the PER and POR could be further dissociated by how a given neuron codes the interaction between the stimulus and response: stimulus-selective neurons of the POR are more strongly modulated by the response factor than neurons of the PER, whereas response-selective neurons of the PER show stronger dissociation for object stimuli compared with scenes.

## Synthesis

Reviewing Editor: Jonathan Lee, University of Birmingham Decisions are customarily a result of the Reviewing Editor and the peer reviewers coming together and discussing their recommendations until a consensus is reached. When revisions are invited, a fact-based synthesis statement explaining their decision and outlining what is needed to prepare a revision will be listed below. The following reviewer(s) agreed to reveal their identity: Rebecca Burwell, Maximiliano Nigro.

The reviewers and reviewing editor agree that your manuscript presents interesting and important new data concerning the relative functions of the perirhinal and postrhinal cortices in visual object and scene processing. The full reviews are appended below and I would ask that you provide a point-by-point response to accompany your revised manuscript. I wish to highlight in particular the reviewer consensus that the introduction and rationale for the study (as well as some of the discussion) is presented within a somewhat outdated conceptual framework that overemphasises the anatomical segregation of inputs to the hippocampus from the PER and POR. Please see the details in each of the sets of reviewer comments.

Reviewer 1 comments:

This is a well written, nicely illustrated paper that reports novel findings about the functions of the perirhinal (PER) and postrhinal (POR) cortices using simultaneous recordings in the two regions in rats performing a goal-directed visual scene and object memory task. They report that PER and POR showed similar proportions of object-selective and scene-selective neurons, but that the more POR neurons showed selectivity for a given choice response.

These findings are important as they provide evidence that theories of hippocampal function based on spatial and nonspatial processing streams have been oversimplified and should be reevaluated.

1) Introduction: Regarding the segregation of inputs, the authors should also take into consideration Doan et al Witter (2019) and Burwell & Amaral (1998, Perirhinal and postrhinal cortices of the rat: interconnectivity and connections with the entorhinal cortex). These papers report that the postrhinal cortex projects to the lateral entorhinal cortex as well as the medial entorhinal cortex.

2) This is a great task for the study, but is it really a memory task? It seems the authors are recording once the animals have learned the discrimination very well. So, is the main memory component the matching of the stimulus to the response? They should comment on this in the discussion.

3) Figure 1B. The authors should make better pictures for response box or use a schematic. It is very hard to tell what I am looking at although maybe the quality will be better at publication.

4) Figure 2. Is the behavior for all 8 rats or just the rats with good tetrodes in figure 3?

5) Line 104. This is a relatively small number of cells, but it is hard to record in these regions. There are tetrode locations for six rats, but were cells recorded from all rats?

6) Line 217. Small numbers can be an issue when comparing frequencies, but using the Fisher Exact test seems appropriate for these numbers, I think that is fine.

Reviewer 2 comments:

My major comment concerns the starting hypothesis that PER and POR represent the origin of two parallel pathways to the hippocampal formation (see comment below). Doan et al. (2019) have demonstrated that the projections of PER and POR converge in LEC. Thus the parallel pathway hypothesis does not seem to be a valid staring point anymore. I suggest the authors to de-emphasize it in the Introduction and Discussion (and add the relevant references for it). The authors may rather focus on the cited behavioral work showing that both areas are related to object and scene processing, however PER has been shown consistently to be involved in object recognition in different species.

• Line 37: this statement is no longer valid. Indeed Doan et al. (2019) recently described how PER and POR projection converge in LEC. POR projects to a very ventral part of MEC which has not been functionally characterized yet.

• Line 795: salient should be silent

• Line 190 and Figure 5: Cell#2 does not seem to decrease its firing rate preferentially during pebble trials. From the raster plot it seems to remain silent until after the choice. Is this cell responding to reward instead?

• Line 225 and onwards: the authors refer to neurons that decrease their firing rates in presence of a particular stimulus as inhibitory. This is a bit confusing and different terminology would be preferred, such as inhibited and excited.

• Line 249: does the expression firing patterns have the same meaning as firing rates? If yes, please use only firing rates, otherwise explain

• Lines 365-381 (Discussion): here the authors reiterate the assumption of parallel pathways in MTL. This was mainly based on anatomical examination of the outputs of PER and POR (Suzuki and Amaral 1994; Naber et al., 1997; Burwell and Amaral 1998; none of which is cited in the manuscript). However, recent extensive examination with antero, and retrograde tracing methods and neurophysiology, demonstrated that the POR targets LEC and not MEC as previously shown (Doan et al., 2019). These new findings invalidate the assumption of parallel pathways for objects and place coding originating in PER and POR. Moreover, responses to objects are found in both LEC (targeted by PER and POR) and MEC, suggesting that object information might enter the parahippocampal formation through more than one pathway.

• Line 414: delete be

## Author Response

We thank both reviewers for their careful reading of our manuscript and constructive

comments. Our point-by-point responses to reviewers’ comments are provided

below.

Reviewer #1

R 1-1. Introduction: Regarding the segregation of inputs, the authors should also

take into consideration Doan et al Witter (2019) and Burwell & Amaral (1998,

Perirhinal and postrhinal cortices of the rat: interconnectivity and connections with

the entorhinal cortex). These papers report that the postrhinal cortex projects to the

lateral entorhinal cortex as well as the medial entorhinal cortex.

: As the reviewer commented (also reviewer 2 had the same comments), Doan

et al. (2019) has provided significant evidence that needs to be taken seriously by

those who have been in favor of the notion that there are parallel anatomical

structures and segregated information processing streams in the MTL. Doan et al.

have shown that the POR as well as the PER project heavily to the LEC, and,

surprisingly, the POR-to-MEC projections are rather sparse, contrary to what has

been known in the literature. We agree with the reviewer that it is important to

mention this and other related critical findings in our introduction and discussion. We

have revised our manuscript to do so and, at the same time, have toned down the

importance of some of the previous studies supporting the segregation of inputs

within the MTL as follows.

”...Traditionally, the PER and POR have been considered to send fairly segregated projections to the lateral

entorhinal cortex (LEC) and medial entorhinal cortex (MEC), respectively (Suzuki and Amaral, 1994; Naber et

al., 1997; Burwell and Amaral, 1998). In support of the anatomical literature, some significant physiological

differences have been reported between the MEC and LEC (Hafting et al., 2005; Savelli et al., 2008; Solstad et

al., 2008; Kropff et al., 2015)...However, recent anatomical findings have shed new light on this notion of

segregated pathways of the MTL by showing that the POR in addition to the PER project heavily to the LEC

(Doan et al., 2019). Furthermore, the POR and PER are interconnected reciprocally (Burwell and Amaral,

1998b; Lavenex et al., 2004; Burke et al., 2018). It is also unclear whether the previous experimental evidence

supports the clear physiological differentiation between the PER and POR (Furtak et al., 2012; Bos et al., 2017).

Also, the PER and POR are not dissociated very well in a visual scene memory task, compared to the LEC and

MEC (Park et al., 2017). Considering these findings, the traditional view needs to be modified to guide future

experimental work and reinterpret the results from prior studies that showed segregated functions of the PER

and POR (Manns and Eichenbaum, 2006; Knierim et al., 2014). For this purpose, the neural correlates of the

PER and POR need to be directly compared within the same animals that perform in a common task, but very

few studies have done so.” (Lines 70-92)

"However, the results from our studies overall suggest that the PER and POR may not always be dissociable

based solely on the characteristics of sensory inputs. Indeed, there is a growing body of evidence now indicating

that there are anatomical and functional overlaps between the so-called spatial and non-spatial information

streams in the MTL (Burke et al., 2018). Doan et al. (2019) reported direct anatomical and physiological

projections from the POR to the LEC. Furthermore, POR neurons mainly responded to the conjunction of an

object and its spatial location, but not to a specific spatial location in the environment (Furtak et al., 2012). In

addition, some PER neurons showed sustained activity to a large patch of the spatial layout (Bos et al., 2017),

and fired in association with spatial locations in the absence of objects (Burke et al., 2012). These findings

support the idea that the PER takes part in spatial information processing. The relatively unclear dissociation

between the PER and POR may also be reflected in the seemingly related findings in their downstream regions.

That is, in the MEC, object-vector cells that code a specific distance and direction information from an object

have been reported (Hoydal et al., 2019), and some LEC neurons could represent the rat’s egocentric bearings to

the boundaries in an open arena even when there was no object cue (Wang et al., 2018). Our results further

emphasize the need for revising the traditional dual-stream model to incorporate dynamic interplays of spatial

and non-spatial coding at multiple levels in the MTL (Connor and Knierim, 2017; Burke et al., 2018; Doan et

al., 2019; Fiorilli et al., 2021).” (Lines 591-608)

R 1-2. This is a great task for the study, but is it really a memory task? It seems the

authors are recording once the animals have learned the discrimination very well.

So, is the main memory component the matching of the stimulus to the response?

They should comment on this in the discussion.

: The reviewer is correct in that we recorded neural activity in well-trained rats.

Therefore, we could not analyze the neural signals related to the acquisition phase.

We also agree that the association between stimulus and response is the key

cognitive component in our task. However, we want to clarify that we intended to test

"recognition memory” for objects and scenes, but not perceptual discrimination of

stimuli. While we utilized a limited number of stimuli, our task cannot be solved if rats

are unable to recognize a given stimulus and compare it against the previously

formed memory to retrieve its associated behavioral response. Therefore, we

respectfully disagree with the reviewer’s comment that there is no memory

component in our task. It requires object or scene “recognition memory” in the first

place.

One direct evidence that our VSOM task requires recognition memory can be

found in our previous study that used a similar behavioral paradigm (Ahn et al.,

2019). In the study, rats were required to choose a push or nose-poke response

based on the capability of recognizing the identities of four different object stimuli

attached to the response box. In this study, PER neurons decreased their firing rates

to repeated exposure to object stimuli, which is one of the most well-known neural

signatures of recognition memory.

We appreciate the reviewer’s comment for giving us a chance to clarify this

critical point of our study, and added a new paragraph in the discussion section as

follows:

"Although we only recorded neural activities in well-trained animals and thus could not monitor the neural

correlates of acquisition, this does not necessarily mean that our task was not a memory task. Successful

performance in our task requires normal object recognition memory. Our previous study has reported the neural

activity often taken as physiological evidence of object recognition (i.e., repetition suppression) in the same

behavioral paradigm in well-trained rats (Ahn et al., 2019).” (Lines 550-555)

R 1-3. Figure 1B. The authors should make better pictures for response box or use a

schematic. It is very hard to tell what I am looking at although maybe the quality will

be better at publication.

: We agree with the reviewer and have replaced Figure 1B with a more detailed

illustration of the response box so that readers can have better views of the specific

components. We also rewrote the figure legend accordingly. We also created Movie

1 to show a rat performing four consecutive trials including push and nose-poke

responses to different stimulus conditions to help readers to understand our task

better.

R 1-4. Figure 2. Is the behavior for all 8 rats or just the rats with good tetrodes in

figure 3?

: We included all eight rats to analyze behavior data as we mentioned in the

methods section of our original manuscript, but neural data were analyzed in six rats

with valid tetrodes in the PER and POR. The other two rats were excluded from

neural data analyses because of the misplacement of the tetrodes in the

intermediate hippocampus, but not in the PER or POR. We have revised our

manuscript to convey more accurate information by mentioning how the two rats

were eliminated in our Methods and also revising our statistical analysis of

behavioral data based on 6 rats (Lines 165-176). The results are identical to those in

the original manuscript. Also, Figure 2 was revised by using six rats but not eight.

R 1-5. Line 104. This is a relatively small number of cells, but it is hard to record in

these regions. There are tetrode locations for six rats, but were cells recorded from

all rats?

: Yes. Except for the two rats excluded from analysis for tetrode misplacement,

cells were recorded from all six rats used in the final analysis. We clarified this in our

revised manuscript as follows:

”...We implanted the hyperdrive to target the PER and POR in eight rats, but due to the difficulty in targeting the

slim band of rhinal cortices, two of the rats ended up with their tetrodes targeting only the intermediate

hippocampus. We excluded those two animals, and all analyses on behavioral and neural data were conducted

by using the remaining six rats. Among six rats, cells were recorded from both PER and POR in four rats and

only from PER in two rats.” (Lines 209-213)

”...As mentioned above, two of the rats did not have tetrode tips in the PER or POR, and were eliminated from

further analyses.” (Lines 240-241)

R 1-6. Line 217. Small numbers can be an issue when comparing frequencies, but

using the Fisher Exact test seems appropriate for these numbers, I think that is fine.

: We agree with the reviewer. We have selected the Fisher’s exact test to

overcome the problem of the small sample size. To check the issue further, we also

tried the chi-square test to compare neuronal proportions between the PER and

POR, which yielded the same statistical trend. Specifically, the overall proportions

were significantly different between the PER and POR (p = 0.027). No significant

difference was found in proportions of scene-selective (p = 0.63) or object-selective

(p = 0.19) neurons. The POR had more response-selective neurons compared to the

PER (p = 0.003).

Reviewer #2

R 2-1. My major comment concerns the starting hypothesis that PER and POR

represent the origin of two parallel pathways to the hippocampal formation (see

comment below). Doan et al. (2019) have demonstrated that the projections of PER

and POR converge in LEC. Thus the parallel pathway hypothesis does not seem to

be a valid starting point anymore. I suggest the authors to de-emphasize it in the

Introduction and Discussion (and add the relevant references for it). The authors

may rather focus on the cited behavioral work showing that both areas are related to

object and scene processing, however PER has been shown consistently to be

involved in object recognition in different species.

: This was the common comment from the two reviewers and please see our

response to Reviewer 1’s comment 1. Briefly, we agree with the reviewer and have

revised our manuscript accordingly. We have marked the changed parts of our

introduction (Lines 70-86) and discussion (Lines 591-608) in red colors, but please

also check our response to reviewer 1 to find these revised paragraphs.

R 2-2. Line 37: this statement is no longer valid. Indeed Doan et al. (2019) recently

described how PER and POR projection converge in LEC. POR projects to a very

ventral part of MEC which has not been functionally characterized yet.

: We agree with the reviewer. Please see our response above.

R 2-3. Line 795: salient should be silent

: We appreciate the reviewer’s careful examination of our manuscript. We

revised the manuscript accordingly.

R 2-4. Line 190 and Figure 5: Cell#2 does not seem to decrease its firing rate

preferentially during pebble trials. From the raster plot it seems to remain silent until

after the choice. Is this cell responding to reward instead?

: We agree that the cell remained silent in response to the pebble condition,

rather than decreased its firing rate. So we modified the expression to “remained

silent” when describing cell #2 in the revised manuscript (Lines 390-393).

With respect to the reviewer’s comment on the reward-related signal of cell

#2, we created a raster plot and spike density function aligned to the choice point to

examine this. However, we could not observe any significant modulation

after the choice was made.

In pebble trials, the cell had more spikes after the choice than before,

but the increased firing rates after the choice were not comparable to the firing rates

for zebra, owl, and phone trials before the choice was made (i.e., during the event

period). In addition, our manuscript focused on the selective firing patterns to

task-related conditions, but the firing rates after the choice point did not show

significant differences between conditions.

R 2-5. Line 225 and onwards: the authors refer to neurons that decrease their firing

rates in presence of a particular stimulus as inhibitory. This is a bit confusing and

different terminology would be preferred, such as inhibited and excited.

: We agree with the reviewer that the term “excitatory” or “inhibitory” may

mislead readers because those neurons may not excite or inhibit other postsynaptic

neurons. The reviewer kindly suggested alternative terminology, but in order to

eliminate misunderstanding, we decided not to use the term “excite” or “inhibit”.

Instead, we decided to use the terms “responsive” and “non-responsive” as those

neurons either responded to a preferred stimulus or not. We reflected this change in

the result section (Lines 427-435) and Table 2.

R 2-6. Line 249: does the expression firing patterns have the same meaning as

firing rates? If yes, please use only firing rates, otherwise explain

: The two expressions were intended to have the same meaning, so we

changed “firing patterns” to “firing rates” throughout our manuscript.

R 2-7. Lines 365-381 (Discussion): here the authors reiterate the assumption of

parallel pathways in MTL. This was mainly based on anatomical examination of the

outputs of PER and POR (Suzuki and Amaral 1994; Naber et al., 1997; Burwell and

Amaral 1998; none of which is cited in the manuscript). However, recent extensive

examination with antero, and retrograde tracing methods and neurophysiology,

demonstrated that the POR targets LEC and not MEC as previously shown (Doan et

al., 2019). These new findings invalidate the assumption of parallel pathways for

objects and place coding originating in PER and POR. Moreover, responses to

objects are found in both LEC (targeted by PER and POR) and MEC, suggesting

that object information might enter the parahippocampal formation through more

than one pathway.

: We agree with the reviewer and have revised our manuscript accordingly. Please

see our response to reviewer #1 (R1-1).

R 2-8. Line 414: delete be

: We have corrected the error.
